# Prevalence of HIV and sexually transmitted and blood-borne infections, and related preventive and risk behaviours, among gay, bisexual and other men who have sex with men in Montreal, Toronto and Vancouver: results from the Engage Study

**DOI:** 10.17269/s41997-021-00546-z

**Published:** 2021-06-17

**Authors:** Trevor A. Hart, David M. Moore, Syed W. Noor, Nathan Lachowsky, Daniel Grace, Joseph Cox, Shayna Skakoon-Sparling, Jody Jollimore, Abbie Parlette, Allan Lal, Herak Apelian, Jordan M. Sang, Darrell H. S. Tan, Gilles Lambert, the Engage Study Team

**Affiliations:** 1 Ryerson University, Toronto, ON Canada; 2 University of Toronto, Toronto, ON Canada; 3 BC Centre for Excellence in HIV/AIDS, Vancouver, BC Canada; 4 Louisiana State University Shreveport, Shreveport, LA USA; 5 University of Victoria, Victoria, BC Canada; 6 McGill University, Montréal, QC, Canada; 7 Direction régionale de santé publique – Montréal, Montréal, QC, Canada; 8 Community-Based Research Centre for Gay Men’s Health, Vancouver, BC Canada; 9 Unity Health, Toronto, Ontario Canada; 10 Centre for Urban Health Solutions, St. Michael’s Hospital, Toronto, Canada; 11 University of Toronto, Toronto, Canada; 12 Institut national de santé publique du Québec, Montréal, Canada

**Keywords:** HIV infections, Sexually transmitted diseases, Sexual behaviour, Sexual and gender minorities, Infections à VIH, maladies transmissibles sexuellement, comportement sexuel, minorités sexuelles et de genre

## Abstract

**Objectives:**

The last Canadian biobehavioural surveillance study of HIV and other sexually transmitted and blood-borne infections (STBBI) among gay, bisexual and other men who have sex with men (GBM) was conducted in 2010. We designed a study to measure STBBI prevalence among GBM in metropolitan Montreal, Toronto and Vancouver and to document related preventive and risk behaviours.

**Methods:**

The Engage Cohort Study used respondent-driven sampling (RDS) to recruit GBM who reported sex with another man in the past 6 months. At baseline, we examined recruitment characteristics of the samples, and the RDS-II-adjusted distributions of socio-demographics, laboratory-confirmed HIV and other STBBI prevalence, and related behaviours, with a focus on univariate differences among cities.

**Results:**

A total of 2449 GBM were recruited from February 2017 to August 2019. HIV prevalence was lower in Montreal (14.2%) than in Toronto (22.2%) or Vancouver (20.4%). History of syphilis infection was similar across cities (14–16%). Vancouver had more HIV-negative/unknown participants who reported never being HIV tested (18.6%) than Toronto (12.9%) or Montreal (11.5%). Both Montreal (74.9%) and Vancouver (78.8%) had higher proportions of men who tested for another STBBI in the past 6 months than Toronto (67.4%). Vancouver had a higher proportion of men who used pre-exposure prophylaxis (PrEP) in the past 6 months (18.9%) than Toronto (11.1%) or Montreal (9.6%).

**Conclusion:**

The three largest cities of Canada differed in HIV prevalence, STBBI testing and PrEP use among GBM. Our findings also suggest the need for scale-up of both PrEP and STI testing among GBM in Canada.

## Résumé

**Objectifs:**

La dernière étude canadienne portant sur la surveillance biocomportementale du VIH et des autres infections transmissibles sexuellement et par le sang (ITSS) chez les hommes gais, bisexuels et autres hommes ayant des relations sexuelles avec des hommes (GBH) a été menée en 2010. Nous avons mis en œuvre une étude visant à estimer la prévalence des ITSS parmi les GBH résidant dans les régions métropolitaines de Montréal, Toronto et Vancouver ainsi qu’à documenter les comportements préventifs et à risque associés à ces infections.

**Méthodes:**

La méthode « d’échantillonnage dirigé par les répondants » (RDS) a été utilisée pour recruter des GBH ayant eu des relations sexuelles avec un autre homme au cours des six derniers mois. Nous avons examiné les distributions, ajustées selon l’approche RDS-II, des caractéristiques socio-démographiques des répondants, et la prévalence du VIH et d’autres ITSS mesurées par des tests de laboratoire ainsi que les comportements associés. Nous avons porté une attention particulière aux différences entre les villes décelées par analyses univariées.

**Résultats:**

Nous avons recruté un total de 2 449 GBH entre février 2017 et août 2019. La prévalence du VIH était plus basse à Montréal (14,2 %) qu’à Toronto (22,2 %) ou à Vancouver (20,4 %) alors que celle de la syphilis (résultat de test compatible avec une infection au cours de la vie) était similaire dans les trois villes (14 à 16 %). Vancouver comptait plus de participants de statut VIH négatif/inconnu ayant déclaré n’avoir jamais passé un test de détection du VIH à vie (18,6 %) comparativement à Toronto (12,9 %) ou à Montréal (11,5 %) alors que les proportions de répondants ayant passé un test de détection d’une autre ITSS au cours des six derniers mois étaient plus élevées à Montréal (74,9 %) et à Vancouver (78,8 %) qu’à Toronto (67,4 %). Vancouver comptait une plus forte proportion d’hommes ayant eu recours à la prophylaxie du VIH pré-exposition (PrEP) au cours des six derniers mois (18,9 %) que Toronto (11,1 %) ou Montréal (9,6 %).

**Conclusion:**

Les trois plus grandes villes du Canada différaient en ce qui concerne la prévalence du VIH, le dépistage des ITSS et l’utilisation de la PrEP au sein des GBH. Nos résultats suggèrent la nécessité d’intensifier le dépistage des ITSS et le recours à la PrEP chez les GBH au Canada.

## Introduction

The last national Canadian biobehavioural STBBI surveillance study of gay, bisexual and other men who have sex with men (GBM), M-Track, was in 2010 (Public Health Agency of Canada [PHAC], 2011). Since that multi-city surveillance study, most biobehavioural Canadian studies focused on GBM have not incorporated cross-city comparisons. As health care in Canada falls under provincial jurisdiction, the prevalence of sexually transmitted and blood-borne infections (STBBI) and related behaviours may differ among provinces with distinct policy environments.

Half of reported HIV diagnoses continue to be among GBM in Canada (Bourgeois et al., 2017). GBM are 131 times more likely to contract HIV than other Canadian men (Yang et al., 2016). HIV prevalence among GBM tends to be highest in the largest cities (e.g., BC Centre for Disease Control, 2019; Blouin et al., 2019; Ontario HIV Epidemiology and Surveillance Initiative, 2018). Similarly, diagnoses of other STBBIs, such as syphilis, chlamydia, gonorrhea, and hepatitis C virus (HCV), remain common among GBM, with GBM comprising the majority of syphilis cases (PHAC, 2019).

UNAIDS behavioural indicators for Global AIDS Monitoring to reduce the burden of HIV and other STBBIs for high-risk populations, such as GBM, include testing for HIV, testing for other STBBIs, using pre-exposure prophylaxis (PrEP) among HIV-negative persons, and reducing condomless anal sex (CAS) with serodiscordant partners (UNAIDS, 2017). In M-Track, 86.2% of GBM reported they had ever tested for HIV, with proportions for other STBBIs being lower, at 62.6–66.9% (PHAC, 2011). No data were available for PrEP as M-Track predated the 2016 Health Canada approval of emtricitabine-tenofovir disoproxil fumarate for use as PrEP (Government of Canada, 2019). In M-Track, 55% of GBM reported CAS in the past 6 months. Contemporary data are needed as studies across Western countries suggest increases in CAS over time (Lachowsky et al., 2016; Holt et al., 2018). Temporal increases in CAS may have taken place quickly in Canada, as Lachowsky et al. (2016) found a significant decrease in condom use at last anal sex act in a 2-year period from 2012 to 2014.

The Canadian government has committed to an integrated focus on ongoing surveillance, testing, prevention, and treatment for STBBIs among those most exposed to infections, including GBM (PHAC, 2019). This approach includes promoting sexual health education, condom use and PrEP. Although there are many similarities across provinces, such as efforts to facilitate early diagnosis and initiation of HIV treatment (Ministère de la Santé et des Services Sociaux, 2020; Montaner et al., 2014; Ontario Clinical Care Guidelines, 2020), there are also significant differences in STBBI policies and programs. Some differences include the early implementation of HIV test-and-treat programs as an official policy in BC since 2010 (Montaner et al., 2014), the relatively early listing of PrEP on the Ontario public formulary in September 2017, and the Montreal community-based HIV testing with peers as HIV counsellors since 2009 (Otis et al., 2016). There are also provincial policy differences in the costs of PrEP. In Québec, PrEP has been offered at low cost, as part of the provincial drug plan (Ministère de la Santé et des Services Sociaux du Québec, 2016). In Ontario, while there is partial public coverage through provincial drug assistance programs (Ontario Ministry of Health and Long-Term Care, 2016), most PrEP is still covered by private insurers (Tan et al., 2020). By contrast, in BC, PrEP has been free of charge for all individuals who meet provincial clinical guidelines since January 2018 (BC Centre for Excellence in HIV/AIDS, 2020).

These provincial differences may result in differences in STBBI prevalence and related behaviours among urban Canadian GBM. In addition, the absence of recent STBBI biobehavioural surveillance limits the ability of policy makers to determine the policies and programs that would have the greatest impact on STBBI control in Canada’s three largest urban centres. Our study provides updated prevalence estimates for HIV and other STBBIs, and related preventive and risk behaviours among GBM. This study of GBM in Montreal, Toronto and Vancouver is called the Engage Cohort Study (Engage). This paper’s objective is to present the study and baseline descriptive statistics on STBBI prevalence and related behaviours as per UNAIDS indicators (2017).

## Methods

Engage combines data from computer-assisted self-interviewing (CASI) and the detection of HIV and other selected STBBIs using biological samples. Engage also involves embedded qualitative studies related to GBM sexual and mental health, blood donation policy and COVID-19 (Grace et al., 2019). Engage was initially designed with a target sample size of *N* = 2160 (*n* = 720 in each city). The Montreal site received supplementary funds from the Québec government to increase their sample size to 1200 participants. Using prevalence data from Vancouver (proportion of CAS with a discordant or unknown HIV status partner among participants in Vancouver was 40% per year; Moore et al., 2016), and assuming a design effect of 2 for the RDS sample (Salganik, 2006), our target sample size of 2160 had 80% power to detect a difference of 6–9% in CAS with a serodiscordant or unknown HIV status partner between cities.

### Community engagement approach

Before initiating recruitment, investigators at each site consulted with Community Engagement Committees (CECs) comprising staff from local community-based organizations focused on GBM health promotion, including AIDS service organizations, and diverse GBM community members in each city. The CECs provided guidance on the content of the questionnaire, recruitment materials and how the study team could effectively communicate findings to GBM communities.

### Sample

Participant eligibility criteria included (1) aged ≥16 years, (2) self-identifying as a man (cisgender or transgender), (3) able to read English or French, (4) live in the metropolitan area of the data collection city, (5) willing to provide biological samples for STBBI testing, and (6) engaged in sexual activity with another man in the 6 months prior to study visit.

### Recruitment via RDS

We used respondent-driven sampling (RDS) to recruit participants in Engage. RDS is a modified form of chain referral sampling designed to approximate probabilistic samples by adjusting for selection bias (Heckathorn, 2002). RDS starts with non-randomly selected initial participants (called “seed participants”) who then continue recruiting eligible members from their social networks until the target sample size is reached.

The study protocol dictated that all sites initiate recruitment with up to 30 seed participants. Monitoring recruitment progress allowed the team to assess the need to add new seeds. The CECs at each site helped to identify initial seed participants from diverse racial/ethnic backgrounds, gender identities, HIV statuses, risk factors for HIV and other STBBIs, and ages. The CECs were the primary source of recommendations for potential seed participants. In Toronto and Vancouver, advertisements on mobile phone applications, such as Grindr, as well as Facebook and Craigslist, were also used to raise awareness of the study and to reach diverse potential seed participants (Lachowsky et al., 2016). After providing written informed consent, seed participants and subsequent participants completed the study questionnaire and biological sampling, and were briefly educated on how to refer other eligible GBM. Each participant received six vouchers to recruit GBM from their social or sexual networks. Participants received CAD$50 compensation for their participation and a secondary incentive of $15 for each eligible GBM they recruited. Each voucher had a unique number to link a participant to his recruiter (thereby establishing recruitment chains and allowing for RDS statistical adjustments) and to facilitate the secondary incentive payment. As per previous RDS literature, we emphasized to seed participants the voluntary nature of recruitment and participation and excluded anyone who indicated that they were coerced into the study. The study was approved by research ethics boards at Ryerson University, University of Toronto, St. Michael’s Hospital, University of Windsor, University of British Columbia, Providence Health Care, University of Victoria, Simon Fraser University, and McGill University Health Centre.

### Measures

#### Biological sampling and STBBI testing

Participants provide a venous blood sample permitting serological testing for HIV, hepatitis C virus (HCV), hepatitis B virus (HBV) and syphilis; these tests were done according to provincial laboratory algorithms, which are similar in the three cities.

Infection with HIV was ascertained using 4th generation testing (detection of HIV antibodies and p24 antigen); reactive results were confirmed using complementary testing (e.g., Western blot analysis). Similar testing was done to confirm HIV infection for participants known to be living with HIV. In Vancouver, participants known to be living with HIV were also offered the option of either confirming their diagnosis with a point of care (INSTI®) test, or by requesting confirmation from their primary care physician.

HCV infection was determined based on a positive HCV antibody result. For men reporting or found to have a current or past HCV infection, HCV RNA testing was also done to evaluate active HCV infection. HBV status was ascertained using testing for hepatitis B surface antigen (HBsAg). We defined active HBV infection as HBsAg positivity.

A history of syphilis infection was based on a positive anti-treponemal antibody test and (1) a rapid plasmin reagin (RPR) titre >1:4, or (2) if the RPR titre was ≤1:4, a reactive *Treponema pallidum* particle agglutination assay (TP-PA) or other treponeme-specific test.

Study participants also provided urine, pharyngeal swabs and rectal swabs. Screening for *Chlamydia trachomatis* and *Neisseria gonorrhoeae* was done using nucleic acid amplification testing (NAAT) or culture, based on provincial laboratory testing procedures. Due to provincial differences, for throat and rectal samples, half of the participants in Toronto had a culture and half had a NAAT, whereas in Montreal and Vancouver, all specimens underwent NAAT testing. For gonorrhea and chlamydia, any positive result on a urine, pharyngeal or rectal specimen was coded as a detected infection.

Test results were made available to study participants within 2 weeks after collection. Staff provided all participants who tested newly positive for HIV or other STBBIs with linkage to local care and treatment providers. The Vancouver site provided treatment for gonorrhea, chlamydia and syphilis on site. Study staff also provided STBBI transmission risk reduction counselling.

#### Questionnaire

We used the Sexual Health Framework (Ivankovich et al., 2013) and the Global AIDS Monitoring preventive and risk behaviour indicators (UNAIDS, 2017) to focus the questionnaire on the societal and community contexts, social relationships, and individual characteristics of sexual health among GBM. For the present analysis, we examined four preventive and risk behaviours: (1) HIV testing, (2) other STBBI testing, (3) PrEP use (continuous or on-demand) in the past 6 months, and (4) engagement in serodiscordant condomless anal sex (SDCAS) at least once in the past 6 months.

### Data analysis

We conducted a cross-sectional analysis of the baseline data collected at study enrolment. We used the RDS-II estimator (Volz & Heckathorn, 2008) for all analyses. Network size was defined as the number of eligible GBM the participant knew who lived or worked in his city (Montreal/Toronto/Vancouver). Examples given to participants included GBM whom the participant sees or speaks with regularly, such as close friends, regular sex partners, and roommates. For the lower limit, we set the minimum value to 1 as participants had to be sexually active with another man in the last 6 months to be eligible for the study. We selected an upper limit of 150, following estimates on the maximum number of possible current relationships (Dunbar, 2010).

For this analysis, we calculated crude point estimates and RDS-adjusted local population-based estimates with 95% confidence intervals (CI) for demographic characteristics, STBBI prevalence, test results and STBBI preventive and risk behaviours. We also examined differences in the RDS-adjusted estimates using non-parametric tests considering unequal variance and sample size across cities. RDS recruitment data were analyzed using RDS Analysis Tool version 7.1 (Volz et al., 2012) and statistical analyses were performed using STATA/SE 13.1 (Statacorp LLC, College Station, Texas, USA).

## Results

### Sample accrual

A total of 27 seeds were recruited in Montreal, 96 seeds in Toronto and 117 seeds in Vancouver. Table 1 presents recruitment characteristics of the sample. Recruitment at all sites started in February 2017 and ended in August 2019. The Montreal sample was recruited in 15 months, the Toronto sample in 27.2 months and the Vancouver sample in 29.8 months. A total of 2449 GBM were recruited: 1179 in Montreal, 517 in Toronto and 753 in Vancouver.

**Table 1:** RDS recruitment characteristics for the Engage Study, 2017–2019 (*N* = 2449)

	Montreal	Toronto	Vancouver
Sample size	1179	517	753
Interview date (first)	07-Feb-17	16-May-17	16-Feb-17
Interview date (last)	15-Jun-18	09-Aug-19	31-Jul-19
Recruitment duration (in days)	493	815	895
Number of seeds, % of sample	27, 2.3%	96, 18.6%	117, 15.5%
Number of productive seeds^1^, % of seeds	21, 77.8%	53, 55.2%	71, 60.7%
Number of RDS coupons distributed	6822	3078	4424
Mean recruitment waves (95% CI)	6.67 (6.45–6.87)	2.67 (2.45–2.89)	2.67 (2.51–2.83)
Mean size of chain (95% CI)	239.38 (230.63–248.13)	29.68 (27.31–32.05)	33.18 (30.75–35.62)
Mean network size^2^ (95% CI)	51.76 (49.01–54.50)	56.78 (52.44–61.13)	53.19 (49.61–56.76)

Note: ^1^Productive seeds indicate participants who recruited at least one eligible person who completed the study; *CI*, confidence interval; ^2^Network size indicates the number of eligible participants (GBM aged 16+ including trans men) the participant knows who live or work in his city (Montreal/Toronto/Vancouver)

### Demographic characteristics

Table 2 presents the crude estimates and the RDS-adjusted estimates of demographic characteristics in each city. We observed differences across cities in demographic characteristics. Montreal GBM were older (RDS-adjusted mean age = 37.9 ± 14.6 [SD]) compared with Toronto GBM (RDS-adjusted mean age = 34.8 ± 12.9; *p* < 0.001) and Vancouver GBM (RDS-adjusted mean age = 35.4 ± 12.6; *p* < 0.001). Toronto GBM were younger than Vancouver GBM (*p* = 0.02). Montreal had a higher proportion of GBM with a high school or less education (25.4%) than Toronto (19.5%, *p* = 0.008) or Vancouver (19.7%, *p* = 0.004). Montreal had a greater proportion of GBM with ≤$30,000 annual income (66.8%) compared with Toronto (57.4%, *p* < 0.001) or Vancouver (61.3%, *p* = 0.01).

**Table 2:** Crude and RDS-II-adjusted distributions of socio-demographic characteristics for each Engage Study site, 2017–2019 (*N* = 2449)

	Montreal (M)		Toronto (T)		Vancouver (V)		City comparison^1^
Variable	Crude	RDS adjusted	Crude	RDS adjusted	Crude	RDS adjusted	
Age, in years, median (IQR)	34 (27–49)	33 (27–49)	31 (27–38)	29 (25–40)	32 (27–43)	31 (25–44)	M > T***; M > V***; V > T*
	%	%	%	%	%	%	
Race/ethnicity							
White	76.9	70.6	64.9	59.9	66.0	54.6	M > T***; M > V***; V = T
Black	2.6	2.2	4.4	5.6	2.6	4.7	T > M***; V > M***; T > V**
Latin American	8.2	10.1	7.7	8.4	7.7	10.4	M = T; M = V; V = T
East-Southeast Asian	1.9	1.9	8.3	10.4	14.7	17.9	T > M***; V > M***; V > T***
Aboriginal	0.8	1.2	0.6	2.2	2.9	3.9	M = T; V > M***; V > T**
South Asian	1.0	2.1	3.9	3.6	2.6	4.7	M = T; V > M**; V = T
West Asian/North African	4.4	7.1	3.1	3.6	0.8	0.4	M > T**; M > V***; T > V***
Unidentified/others	1.7	2.9	3.4	2.9	0.9	0.8	M = T; M > V**; T > V**
Mixed race/ethnicity	2.4	1.9	3.5	3.9	1.6	1.9	T > M*; M = V; T > V*
Education level							
High school or less	20.7	25.4	10.6	19.5	14.4	19.7	M > T**; M > V**; V = T
Some college	34.9	34.9	31.6	37.5	34.3	31.2	M = T; M = V; T > V*
Bachelor degree and above	44.4	39.7	57.8	42.9	51.3	49.2	M = T; V > M***; V > T*
Annual income in CAD							
Less than $30K	57.5	66.8	47.8	57.4	45.5	61.3	M > T***; M > V*; V = T
$30K–$59.9K	30.7	25.3	30.9	31.9	29.6	25.6	T > M**; M = V; T > V*
$60K or more	11.7	7.8	21.3	10.6	24.8	13.1	M = T; V > M***; V = T
Sexual identity:							
Gay	82.1	76.5	77.9	72.4	85.8	79.8	M = T; M = V; V > T**
Bisexual	8.5	12.8	4.4	13.6	5.4	11.5	M = T; M = V; V = T
Queer	5.4	4.6	14.5	9.3	5.8	3.7	T > M***; M = V; T > V***
Pansexual	2.3	3.3	2.5	2.9	1.5	1.1	M = T; M > V**; T > V*
Two-Spirit	0.6	0.5	0.6	1.9	0.8	2.8	T > M**; V > M***; V = T
Others	1.1	2.3	0	0	0.7	1.1	M > T***; M = V; V > T*
Country of birth							
Born outside Canada	30.4	34.8	39.5	42.6	35.9	42.7	T > M**; V > M***; V = T
Born in Canada	69.6	65.1	60.5	57.4	64.1	57.3	M > T**; M > V***; V = T
Marital status							
Married/common law	19.4	17.9	23.1	27.1	20.7	15.9	T > M***; M = V; T > V****
Single/separated/divorced/widowed	80.6	82.0	76.9	72.9	79.3	84.1	M > T***; M = V; V > T****

Note: *RDS*, respondent-driven sampling; *IQR*, inter-quartile range; *CAD*, Canadian dollar

^1^*p*-value for Mann-Whitney *U* test for continuous variables and Z-test of proportions for categorical variables

*p < 0.05; **p < 0.01; ***p < 0.001

### STBBI prevalence by city

Table 3 presents the crude estimates and the RDS-adjusted estimates of STBBI testing history and prevalence in each city. HIV prevalence was lower in Montreal (RDS-adjusted percentage = 14.2%) than in Toronto (22.2%, *p* < 0.001) or Vancouver (20.4%, *p* < 0.001). History of syphilis infection ranged from 14.4% to 15.9% across cities and there were no significant differences (*p* > 0.05). In Vancouver, the rectal swab gonorrhea and chlamydia rectal results were not available for 20% of Vancouver participants. Vancouver GBM had a lower prevalence of detected gonorrhoea at any anatomic site (2.1%) than did Toronto (9.1%, *p* < 0.001) or Montreal GBM (5.5%, *p* = 0.001). Chlamydia prevalence ranged between 3.1% and 5.8%, and Vancouver GBM had a higher prevalence of detected chlamydia at any site (5.8%) than Montreal GBM (3.1%, *p* = 0.006). Hepatitis C antibody seroprevalence was greater among Montreal (7.3%) than Toronto GBM (3.9%, *p* = 0.008) but not more than Vancouver GBM (6.0%, *p* = 0.27); the difference between Toronto and Vancouver was not significant. The prevalence of hepatitis B surface antigen positivity was greater in Toronto (1.3%) than Vancouver GBM (0.3%, *p* = 0.04), but not more than Montreal GBM (0.6%, *p* = 0.36).

**Table 3:** Crude and RDS-II-adjusted distributions of HIV, STI and risk characteristics for the Engage Study, 2017–2019 (*N* = 2449)

	Montreal (*n* = 1179)		Toronto (*n* = 517)		Vancouver (*n* = 753)		City comparison^1^
Variable	Crude	RDS adjusted	Crude	RDS adjusted	Crude	RDS adjusted	
	%	%	%	%	%	%	
HIV and other STBBI test results							
HIV test^2^ results							
Negative	81.8	85.8	80.6	77.8	82.4	79.6	M > T***; M > V***; V = T
Positive	18.2	14.2	19.4	22.2	17.5	20.4	T > M***; V > M***; V = T
Other STBBI test^3^ results							
Syphilis	17.6	14.4	17.4	15.8	15.9	14.5	M = T; M = V; V = T
Gonorrhea	6.9	5.5	9.0	9.1	4.2	2.1	T > M*; M > V**; T > V***
Chlamydia	4.2	3.1	10.5	5.3	8.9	5.8	M = T; V > M**; V = T
Hepatitis C	5.8	7.3	1.8	3.9	3.2	6.0	M > T**; M = V; V = T
Hepatitis B	0.6	0.6	0.6	1.3	0.4	0.3	M = T; M = V; T > V*
Testing behaviours HIV testing							
Never^4,5^	7.5	11.5	5.5	12.9	8.5	18.6	T = M; V > M***; V > T*
Tested within P6M^6^	53.7	52.5	62.3	54.3	68.7	61.9	M = T; V > M***; V > T*
Other STBBI Testing^7^							
Never^5^	11.0	17.9	6.9	17.5	7.9	12.9	T = M; M > V**; T > V*
Tested within P6M	75.8	74.9	81.2	67.4	84.0	78.8	M > T**; V = M; V > T***
Preventive and risk behaviours							
PrEP use, P6M^4^	12.8	9.6	21.2	11.1	24.1	18.9	T = M; V > M***; V > T***
Number of male anal sex partners,P6M, median (IQR)	3 (1–8)	2 (1–5)	4 (1–14)	2 (1–5)	4 (1–8)	2 (1–5)	T > M***; V > M**; T > V*
Any SDCAS, P6M	49.5	42.5	54.9	36.9	53.9	40.1	M > T*; M = V; T = V

Note: *RDS*, respondent-driven sampling; *M*, mean; *SD*, standard deviation; *P6M*, past 6 months; *SDCAS*, serodiscordant condomless anal sex; *IQR*, inter-quartile range

^1^*p*-value using crude estimates for Mann-Whitney *U* test for continuous variables and Z-test of proportions for categorical variables

^2^HIV positive: positive 4th generation testing (detection of HIV antibodies and p24 antigen), confirmed using complementary testing (e.g., Western blot analysis)

^3^Syphilis: history of syphilis infection based on a positive anti-treponemal antibody test and (1) a rapid plasmin reagin (RPR) titre >1:4, or (2) if the RPR titre was ≤1:4, a reactive Treponema pallidum particle agglutination assay (TP-PA) or other treponeme-specific test; Chlamydia and gonorrhea: a positive nucleic acid amplification (NAAT) test or culture, for any of the urine, pharyngeal and rectal specimens. Due to provincial differences, for throat and rectal samples, half of the participants in Toronto had a culture and half had a NAAT, whereas in Montreal and Vancouver, all specimens underwent NAAT testing; hepatitis C: positive HCV antibody result; hepatitis B: positive hepatitis B surface antigen (HBsAg) result

^4^HIV negative/unknown participants only

^5^Never category included never tested, do not remember, and do not know if ever tested

^6^HIV negative/unknown participants who were tested at least once

^7^Self-report of testing for any sexually transmitted infection (STI) other than HIV (including chlamydia, gonorrhea, syphilis, LGV (lymphogranuloma venereum), hepatitis A, hepatitis B, hepatitis C, genital or anal warts, *Shigella*, giardia, herpes (HSV), bacterial vaginosis)

*p < 0.05; **p < 0.01; ***p < 0.001

### STBBI preventive and risk behaviours by city

Regarding HIV testing among self-reported HIV-negative/unknown participants, Vancouver GBM had the highest proportion of participants (18.6%) who reported never being tested compared with Toronto (12.9%, *p* = 0.01) and Montreal GBM (11.5%, *p* < 0.001). Among participants who reported being tested at least once in their lifetime, Vancouver GBM also had the highest proportion of participants (61.9%) who reported being tested in the past 6 months compared with Toronto (54.3%, *p* = 0.02) and Montreal GBM (52.5%, *p* < 0.001). Montreal (17.9%, *p* = 0.003) and Toronto GBM (17.5%, *p* = 0.02) had a greater proportion of participants who had never been tested for an STBBI other than HIV compared with Vancouver GBM (12.9%). Montreal (74.9%, *p* = 0.002) and Vancouver GBM (78.8%, *p* < 0.001) were more likely to have had participants who had been tested for an STI within the past 6 months compared with Toronto GBM (67.4%).

Vancouver GBM (18.9%) were more likely to report PrEP use in the last 6 months than Toronto GBM (11.1%, *p* < 0.001) and Montreal GBM (9.6%, *p* < 0.001). Toronto GBM reported a lower proportion of SDCAS at least once in the past 6 months (36.9%) than Montreal GBM (42.5%, *p* = 0.03). However, we did not see any difference between Montreal and Vancouver GBM (42.5% vs. 40.1%; *p* = 0.30) or between Toronto and Vancouver GBM (36.9% vs. 40.1%, *p* = 0.25).

## Discussion

In this first cross-city biobehavioural comparison of GBM in Canada in over 10 years, we found differences in HIV and other STBBI prevalence, and in related preventive and risk behaviours. HIV prevalence was significantly lower (14.2%) in Montreal than in Toronto (22.2%) or Vancouver (20.4%). Vancouver GBM were more likely to have been tested for HIV within the past 6 months, but were also more likely to report never having been tested for HIV compared with GBM in Montreal and Toronto. Vancouver’s higher proportion of never-tested GBM was unexpected given efforts such as the STOP AIDS initiative and Treatment as Prevention® since 2010 (e.g., Montaner et al., 2014). These findings suggest the need to intensify HIV testing programs for the minority of GBM who have not accessed HIV testing.

The results here also provide current data on STBBI-related needs of GBM across cities beyond HIV. Testing prevalence in the past 6 months for STBBIs other than HIV for Engage is not markedly higher than previous estimates of testing for individual STBBIs (PHAC, 2011). Regarding STBBI testing, Montreal (74.9%) and Toronto GBM (67.4%) were less likely to report having been tested in the past 6 months for an STBBI other than HIV compared with Vancouver GBM (78.8%). The proportion of GBM with a history of syphilis infection ranged from 14% to 16% across cities. The consistency in syphilis antibody prevalence across sites was surprising given city differences in HIV diagnoses, and the high frequency of syphilis/HIV coinfection (e.g., Gesink et al., 2014). Our findings are consistent with a widely recognized resurgence of syphilis among GBM from 2004 to 2015 in many Western countries (Abara et al., 2016). Our prevalence findings are consistent with other studies showing that GBM are disproportionately at risk for STBBI, especially HIV and syphilis (11.2 syphilis cases/100,000 in the Canadian general population; PHAC, 2020). The 13–18% of GBM who had never been tested for an STBBI other than HIV, combined with the syphilis history prevalence in our sample, suggests the need for increased efforts to reach GBM for STBBI-related health services beyond HIV. Hepatitis testing among GBM should be a continued priority, given that 3.9–7.3% have evidence of current or prior hepatitis C virus infection and that 0.3–1.3% have active hepatitis B virus infection in the current study.

Regarding preventive and risk behaviours, PrEP use in the past 6 months among HIV-negative GBM was 9.6% in Montreal, 11.4% in Toronto and 18.9% in Vancouver. It should be noted that the Montreal site completed data collection before the Toronto and Vancouver sites, which may have led to PrEP use being slightly lower in Montreal as PrEP use has continued to scale up in Canada following federal approval in 2016 (Mosley et al., 2018). PrEP use among HIV-negative GBM should be significantly higher in order to reduce HIV transmission as per clinical guidelines (Tan et al., 2017). However, across all cities, SDCAS in the past 6 months was relatively common (36.9–42.5% of GBM). Given the higher proportion of GBM in Vancouver engaging in SDCAS and greater PrEP use, focused efforts on testing and treating PrEP users for STBBI beyond HIV continue to be important.

### Strengths and limitations

Our study represents GBM from the three geographic areas of highest HIV prevalence in Canada. Thanks to the RDS design (Heckathorn, 2002), the estimates presented here may be more accurate than in convenience samples due to our ability to correct for selection biases by adjusting estimates. Engage also extends previous research by combining self-report with biological measurement and the use of consistent measures across the three largest cities in Canada.

We should note that the data here present lifetime syphilis infection and lifetime hepatitis C infection as opposed to current infection. The use of throat and rectum culture testing for gonorrhea/chlamydia for half of the Toronto sample, and the missing data for 20% of rectal samples in Vancouver may have underestimated prevalence for these STIs in these two cities. Montreal’s longer RDS recruitment chains may also have produced a more generalizable sample, including men who may have been less likely to have been reached in other studies. The differences in length of recruitment period between sites may have also led to biases in STBBI point prevalence estimates. We also note that while these data may approximate a probabilistic sample because estimates have been adjusted for RDS recruitment, the data are not adjusted for other factors such as socio-demographic or temporal differences (e.g., syphilis outbreaks), which could affect STBBI comparisons across cities.

Methodologically, our requirement for participants to complete STBBI testing may have underrepresented those who are not interested in getting tested. The smaller number of seed participants and larger sample size of the Montreal sample may have led to more reliable estimates in Montreal compared with Toronto or Vancouver, including the lower HIV prevalence found in Montreal. Given that provinces enacted HIV-related policies at different times and with different levels of access, it is unknown whether there is a need to focus programs differently across cities. Many of the outcomes assessed will therefore require further analyses and more detailed modelling, such as examining how HIV prevalence differences could be explained by differences in age or race across the three samples.

## Conclusion

Engage is the first study in approximately a decade to present HIV and other STBBI prevalence using biological data in a representative sample of urban GBM in Canada. We identified some similarities in sexual health outcomes among GBM in the largest cities in Canada such as history of syphilis infection, but also notable differences, such as in HIV prevalence and PrEP uptake. It is essential to scale up both PrEP and STBBI testing in order to impact HIV and STBBI transmission among Canadian GBM.

### Contributions to knowledge

What does this study add to existing knowledge?

- The last national Canadian biobehavioural sexually transmitted and blood-borne infection (STBBI) surveillance study of gay, bisexual and other men who have sex with men (GBM) was in 2010 (Public Health Agency of Canada, 2011).
- This study examines differences among the 3 largest cities in Canada in HIV and other STBBI prevalence, STBBI testing, and use of HIV pre-exposure prophylaxis (PrEP) among GBM.
- Across cities, we found a high prevalence of GBM with a history of syphilis infection. We found city differences in HIV prevalence, serodiscordant condomless anal sex, and use of pre-exposure prophylaxis.

What are the key implications for public health interventions, practice or policy?

- Our prevalence findings are consistent with other studies showing that GBM are disproportionately at risk for STBBI, especially HIV and syphilis.
- Differences found across cities suggest the need for focused efforts by city.
- These data suggest that increasing STBBI testing and treating PrEP users for STBBI beyond HIV continue to be important. It is essential to scale up both PrEP and STBBI testing in order to impact HIV and STBBI transmission among Canadian GBM.

## Data Availability

Deidentified participant data used in this analysis are stored at the local site where analyses were conducted and the BCCfE. For information regarding these databases, and related access, please contact Engage senior author on manuscript, ORCID ID https://orcid.org/0000-0001-5107-7452, a principal investigator on the Engage Study.
